# Potential Application of T-Follicular Regulatory Cell Therapy in Transplantation

**DOI:** 10.3389/fimmu.2020.612848

**Published:** 2021-02-02

**Authors:** Caroline Dudreuilh, Sumoyee Basu, Cristiano Scottà, Anthony Dorling, Giovanna Lombardi

**Affiliations:** ^1^ Department of Inflammation Biology, King’s College London (KCL), Guy’s Hospital, London, United Kingdom; ^2^ Centre for Nephrology, Urology and Transplantation, School of Immunology and Microbial Sciences, King’s College London, London, United Kingdom; ^3^ NIHR Biomedical Research Centre-Transplant Theme, Guy’s Hospital, London, United Kingdom; ^4^ Peter Gorer Department of Immunobiology, School of Immunology and Microbial Science, King’s College London (KCL), Guy’s Hospital, London, United Kingdom

**Keywords:** regulatory T cell, T-follicular regulatory cell, transplantation, cell therapy, immunosuppression

## Abstract

Regulatory T cells (Tregs) constitute a small proportion of circulating CD4^+^ T cells that function to maintain homeostasis and prevent autoimmunity. In light of their powerful immunosuppressive and tolerance-promoting properties, Tregs have become an interesting potential candidate for therapeutic use in conditions such as solid organ transplant or to treat autoimmune and inflammatory conditions. Clinical studies have demonstrated the safety of polyclonally expanded Tregs in graft-versus-host disease, type 1 diabetes, and more recently in renal and liver transplantation. However, Tregs are heterogenous. Recent insights indicate that only a small proportion of Tregs, called T follicular regulatory cells (Tfr) regulate interactions between B cells and T follicular helper (Tfh) cells within the germinal center. Tfr have been mainly described in mouse models due to the challenges of sampling secondary lymphoid organs in humans. However, emerging human studies, characterize Tfr as being CD4^+^CD25^+^FOXP3^+^CXCR5^+^ cells with different levels of PD-1 and ICOS expression depending on their localization, in the blood or the germinal center. The exact role they play in transplantation remains to be elucidated. However, given the potential ability of these cells to modulate antibody responses to allo-antigens, there is great interest in exploring translational applications in situations where B cell responses need to be regulated. Here, we review the current knowledge of Tfr and the role they play focusing on human diseases and transplantation. We also discuss the potential future applications of Tfr therapy in transplantation and examine the evidence for a role of Tfr in antibody production, acute and chronic rejection and tertiary lymphoid organs. Furthermore, the potential impact of immunosuppression on Tfr will be explored. Based on preclinical research, we will analyse the rationale of Tfr therapy in solid organ transplantation and summarize the different challenges to be overcome before Tfr therapy can be implemented into clinical practice.

## Introduction

Over the last three decades, despite an improvement in short-term outcomes after solid organ transplantation, long-term outcomes have not drastically improved (1). There has been a massive leap in understanding the mechanisms that cause chronic allograft dysfunction (CAD) leading to graft loss, particularly in kidney transplantation. Immune mediated injury is the predominant cause of CAD, many cases of which are associated with the presence of donor specific antibodies (DSA), directed predominantly against donor human leukocyte antigens (HLA). These DSA are produced by B cells in response to alloantigen stimulus through a process that is T-cell regulated (2). The majority of traditional therapeutic strategies attempted have focussed on either targeting B cells or whole T cell population or on removing the DSA themselves, without more precise targeting. Targeting the immune allo-responses to regulate DSA responses might be a way to improve patient outcomes.

CD4^+^ T cells play an important role in both activating other cells (including B cells) and in regulating the immune response (regulatory T cells-Tregs) (3). The old concept of T cell ability to help B cells has been further clarified recently with the discovery of a new small subpopulation of CD4^+^ T cells, called T follicular helper cells (Tfh) (4–6). Tfh are responsible for the interactions with B cells in the germinal centers (GC) within the secondary lymphoid organs (SLO) (spleen or lymph nodes) (7). They support B cells in the process of antibody production (4). In addition, Tfh help in promoting the differentiation of B cells into memory B cells and long-lived plasma cells (8). Human and murine Tfh display similarities in phenotype (expressing CD4, CXCR5, PD1, and Bcl6; lacking expression of CCR7 and IL-7Rα and secreting IL-21) (9–11) and function, responsible of interaction and activation of GC B cells leading to antibody production.

Tfh display a Treg counterpart population called T follicular regulatory cells (Tfr), which have the role of regulating specific interactions between B cells and Tfh (12–14). Tfr have been extensively studied in mouse models, with fewer studies in humans, due to the challenges of sampling human SLO. In human, they have been characterized as being Tfh-like [expressing CD4, the C-X-C chemokine receptor type 5 (CXCR5) and Bcl-6 in association with CD25 and FOXP3] and express varying levels of PD-1 and ICOS depending on their localization. They can be found in SLO like tonsil, lymph nodes, spleen, and ectopic lymphoid structures and even in blood (bTfr) (15). The bTfr remain to be further characterized in humans and their function remains poorly understood. In general, while the role of Tfr in modulating autoimmune responses seems to be crucial, the exact role of these cells in the transplantation settings remains to be elucidated. Here, we review the current evidence around the origin of Tfr, the different subtypes and associated functions. We will discuss the role of Tfr in human diseases with a focus on transplantation and explore the potential of cell therapy using Tfr.

## Definition and Origin of Tfr

### Definition of Tfr and Tfh With Focus on the Differences Between Mouse and Human Tf Cells

The discovery that only a small fraction of CD4^+^ T cells was involved in antibody production (Tfh) and modulation (Tfr) within the GC is recent. Interestingly, although the role of Tregs in regulation of the GC response was first described more than fifteen years ago in human (16, 17), the formal discovery of the Tfr subtype occurred later in murine models (12–14). Tfr are proposed to form when FOXP3^+^ precursors acquire a Tfh-like phenotype that includes expression of Bcl-6, CXCR5, PD-1, and ICOS. Although Tfr share some Tfh features, they do not express/produce the same cytokines which characterize Tfh, such as IL-21, IL-4. In addition to FOXP3, Tfr cells express the typical markers expressed by Tregs such as GITR, Blimp-1, and CTLA-4 (12–14).

Precise definition of Tfr as a sole entity is complicated by the dynamic expression of some chemokine receptors, particularly as the cells navigate between compartments within the SLO, and within the body (see section on *Maturation of Tfr*), as they can be found in blood and SLO like tonsil, lymph nodes, spleen and ectopic lymphoid structures. However, distinguishing between Tfh and/or Tfr with only one specific marker is almost impossible. Most of the work on these two subtypes of T cells has been done in mouse models with this work inevitably influencing study of human counterparts. CXCR5 is probably the most accepted and used marker for Tfh and Tfr (12–14). CXCR5 is a G protein receptor for the chemokine CXCL13. It enables T cells to migrate to the B cell zones of the lymph nodes. This is supported by the evidence that CXCR5 knock out mice present a complex pattern of lymph node developmental defects and a completely disorganized splenic microarchitecture, lacking segregated T- and B-cell areas (18). However, it has been demonstrated recently that some Tfr can access the GC independently of CXCR5 (19), and that after interaction with B cells they proliferate less than their CXCR5^+^ counterparts. Interestingly CXCR5 expression seems to be regulated by nuclear factor of activated T cells 2 (NFAT2) in Tfr (20) but by achaete-scute homologue-2 (ASCL2) in Tfh (21). The involvement of NFAT2 regulation in Tfr was confirmed in Nfat2fl/fl x Cd4cre mice by the demonstration that NFAT2 knock out mice displayed reduced numbers of Tfr (20).

Until recently, studies describing CD4^+^CXCR5^+^ cells did not differentiate between Tfh and Tfr (4, 7, 11). It is not clear what proportion of the CD4^+^ CXCR5^+^ population are Tfr cells. A recent report suggested that CD4^+^CXCR5^+^FOXP3^+^ cells accounted for only 12.8% of circulating CD4^+^ CXCR5^+^cells (22), implying that the majority of circulating CD4^+^CXCR5^+^cells were Tfh, although without confirmatory evidence of a more detailed phenotypic characterization (15).

**Figure 1:**
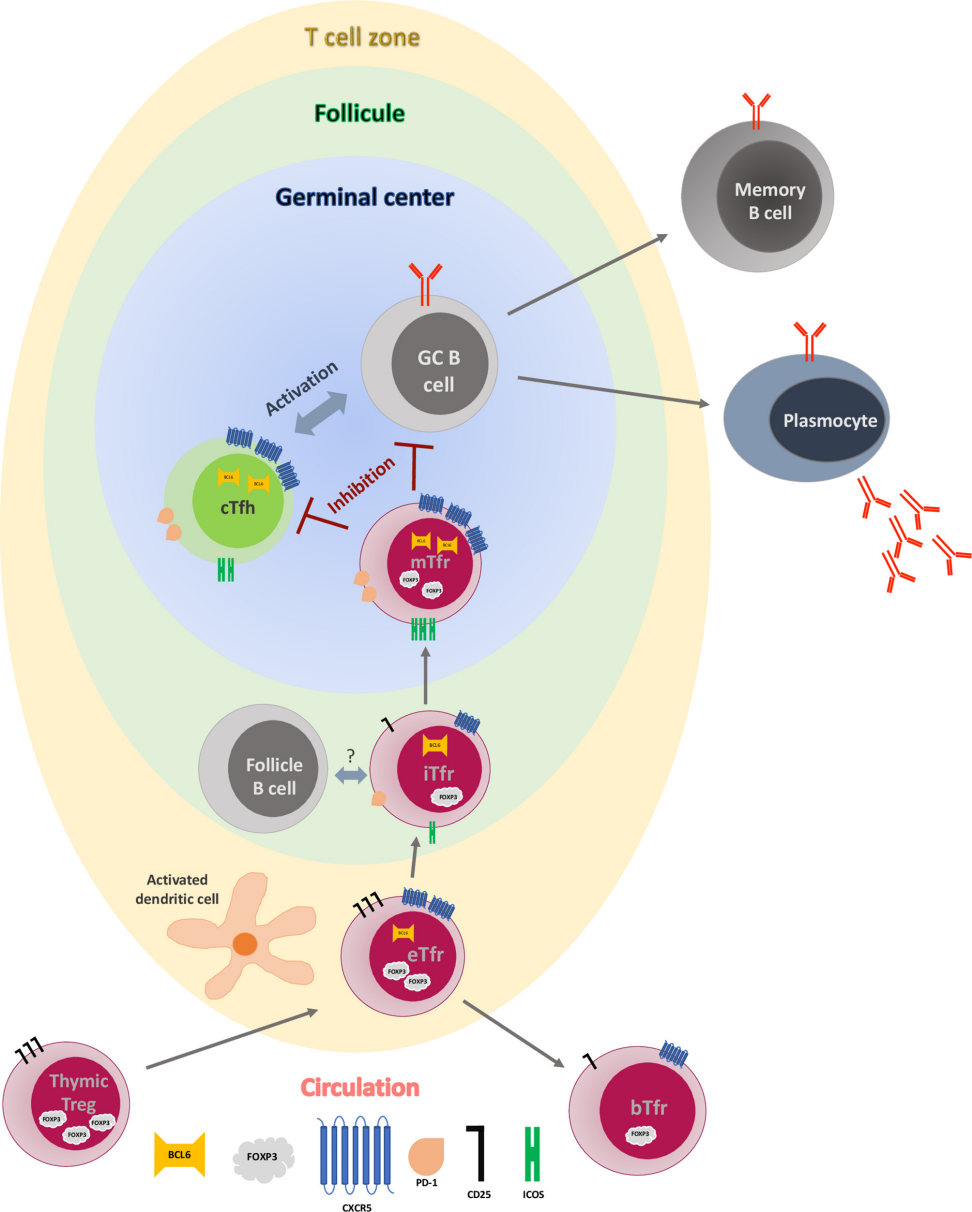
Early germinal center reaction and T follicular regulatory cell maturation process. Early Tfr (eTfr) derive from natural regulatory T cells (Tregs) after expression of CXCR5 and Bcl-6 and down regulation of FOXP3. After interaction with activated dendritic cells (DC) in the T cell zone, some eTfr lose expression of Bcl-6 and enter the circulation (blood Tfr-bTfr), while some migrate to the follicle (intermediate Tfr-iTfr), where they interact with the follicular B cells and start expressing PD-1 and ICOS. Eventually, they move to the germinal center (GC), becoming mature Tfr (mTfr), where they inhibit both the central T follicular helper cell (cTfh) and the GC B cells, leading to regulation of antibody production and B cell differentiation.

Current evidence suggest that Tfr derive from Tregs, at least in mouse (see below), and represent 18.57±6.55% of the total CD25^+^FOXP3^+^ T cells (23). Therefore, FOXP3 is expressed in Tfr at different levels through their differentiation process (see **Figure 1**). Another way to identify Tfr would be the use of the combination of markers CD4^+^CD25^high^ CXCR5^+^CD127^low^, as circulating Tfr are CD25^high^CD127^low^ as they originate from Tregs, while blood Tfh express heterogenous amount of CD25 (negative to low) and a low level of CD127 (24).

The phenotype and function of Tfr and Tfh both depend on the expression of Bcl-6 and STAT3 (5, 12, 14, 25, 26). However, only Tfr express Bcl-6 alongside the Bcl-6 antagonist Blimp-1, although this has only been demonstrated in mouse and not yet in human Tfr (23). While Bcl-6 is important for the Tfh-like properties of Tfr, Blimp-1 is associated with the Treg-like phenotype and function of Tfr (12, 27–29). Blimp1 is a transcriptional repressor protein that suppresses Bcl6 expression. Tfr numbers are regulated through a balance between Bcl-6 and Blimp-1 (12). IL-2 is a key factor regulating Tfr differentiation, promoting Blimp1 expression while repressing Bcl6 in Tregs to preclude Tfr cell development (30).

In humans, they have been characterized as being CD4^+^CD25^+^FOXP3^+^CXCR5^+^ cells with different levels of PD-1 and ICOS expression depending on their localization (31, 32) (and on the transcription factor Bcl-6 for differentiation and localization into the B cell follicle (4–6, 14, 33). ICOS expression in human does not discriminate Tfr from other Tregs (34).

As none of the individual markers described above seems to be specific for Tfh/Tfr and blood, a combination of surface markers is necessary to be able to characterize these two subsets properly, from their origin to the fully matured T cells (see **Table 1** and **Figure 1**). The function of Tfr in a normal immunological response is described below.

**Table 1:** Different expression of markers in different types of Tfr and Tfh compared to Tregs and T naive.

	T Naive	bTfh (blood)	cTfh (central GC)	Treg	eTfr	bTfr	iTfr	mTfr	Ref
**CD4**	++	++	++	++	++	++	++	++	(8, 11–15, 24, 31, 35)
**CXCR5**	-	+++	+++	-	++	++	++	+++	(8, 11–15, 24, 31, 35, 36)
**CD25**	-	-	-	+++	+++	+++	+	–	(8, 11–15, 24, 31, 35)
**FOXP3**	-	-	-	++	++	++	++	++	(8, 11–15, 24, 31, 35)
**ICOS**	+	-	++	+	?	-	++	+++	(8, 11, 12, 15, 24, 31, 35, 36)
**PD-1**	-	-	++	±	–	-	+	++	(8, 11–15, 24, 31, 35)
**Bcl6**	-	-	++	-	+	-	+	++	(8, 11–13, 15, 24, 35)
**Blimp1**	-	-	-	+	+	?	+	+	(8, 11, 12, 15, 24, 31)
**CTLA-4**	-	+	?	+++	?	+++	+++	+++	(12, 15, 37)

Expression of different surface markers (CD4, CXCR5, CD25, ICOS,PD-1) and intracellular markers (FOXP3, Bcl-6, Blimp-1, CTLA-4) in different subtypes of T cells: Naïve T cell, blood T follicular helper cells (bTfh), central Tfh (cTfh), regulatory T cells (Tregs), early T follicular regulatory cell (eTfr), blood Tfr (bTfr), intermediate Tfr (iTfr), and mature Tfr (mTfr). The markers described have been identified in both mouse and human (dark gray), in mouse only (light gray), or hypothesized in the publication from Fonseca et al., in Immunol rev 2019 (white). Of note, “bTfr” refers to blood Tfr, and “cTfr” to circulating Tfr as indicated in some publications (7, 15). mTfr refers to mature Tfr and these subtype of Tfr has been described within the germinal centre.

“-” refers to no expression, “+” refers to low expression, “++” refers to intermediate expression, and “+++” refers to high expression.

### Germinal Center Reaction

GC are defined structures that develop within the SLO during ongoing immune responses; they have been extensively studied and described in mouse models. Through a process called GC reaction, a naïve mature B cell first undergoes clonal expansion and somatic hypermutation within the dark zone of the GC before moving to the light zone (38). There, B cells demonstrating a relevant affinity toward the antigen of interest form cognate interactions with Tfh cells primed by the same antigen. These Tfh help promote B cell responses by providing cytokines (such as IL-21, IL-4) and co-stimulation (through the inducible costimulatory molecules ICOS and CD40L) (7, 35, 39–41). Continued cognate interaction between Tfh and B cells drives immunoglobulin class switching, somatic hypermutation, and B cell differentiation (42) leading to the production of long-lived plasma cells and memory B cells.

### Mechanisms of Regulation by Tfr and Antibody Production

In adoptive transfer experiments of Tfr and Tfh in mice lacking these population of cells, the group of Sage et al. have demonstrated that Tfr have a direct impact on B cell effector function by decreasing antibody secretion, and inhibiting somatic hypermutation and class-switch recombination (23, 31, 36). However, Tfr can also act indirectly to inhibit antibody responses by specifically suppressing production of IL-4 and IL-21 by Tfh, leaving other functions intact (36).

Different approaches have been used to address the specific role of Tfr in regulation of GC responses. The first series of reports used Bcl-6 as a surrogate marker of Tfr in genetic and/or bone marrow chimera models. In these models, Bcl-6 was deleted in FOXP3^+^ cells. The results were contradictory, with some studies indicating that Bcl-6 was essential for Tfr function (12, 43). Fu et al., for example, using Bcl-6fl/flFOXP3Cre (KO) mice, which have reduced numbers of CXCR5^+^PD1^+^CD4^+^FOXP3^+^ Tfr cells, demonstrated enhanced protection against influenza virus associated with an increase in humoral autoimmunity (43). Others, however have demonstrated that the lack of Bcl-6^+^FOXP3^+^ cells did not impact on the development of the Tfh-cell population and numbers of GC B cells, but did alter the levels and avidity of the antigen-specific IgG response (44).

As described earlier, Bcl-6 is not an absolute marker of Tfr and could be expressed by other Tregs. Therefore, Clement et al. (45), designed an inducible Tfr cell-deletion model aiming to study the role of Tfr in an intact host. They generated a strain of mice called T_FR_-DTR (for Diphtheria Toxin receptor) where DTR expression is under the control of a recombinant *Cxcr5* gene in cells expressing FOXP3. Thus, only Tfr cells expressing both FOXP3 and CXCR5 expressed DTR on their surface so were susceptible to deletion by DT. After immunization with (4-hydroxy-3-niotrophneyl)acetyl-ovalbumin (NP-OVA) Tfr in these mice regulated only early GC responses to antigen-specific antibody and B cell memory. Lack of Tfr was associated with a surge of self-reactive IgG and IgE, demonstrating a key role of Tfr in preventing these potentially deleterious responses post-vaccination. Both blood Tfh (bTfh) (46) in HIV+ patients, and bTfr seem to have a memory function and able to be redirected toward antigen re-exposition in other diseases (8, 36).

Although the mechanisms of regulation by Tfr *in vivo* are still under investigation and what is known has been reviewed recently (23, 47, 48), a summary of some of the most important functions of Tfr is set out below. CTLA-4 expression by Tfr has been shown to be key for their function, as conditional deletion of CTLA-4 on Tfr inhibits their function, GC B cells are not inhibited and this leads to increased amounts of antibody produced (47). Furthermore, Tfr cells inhibit antigen-specific IgG levels when adoptively transferred into CTLA-4 inducible knockout (KO) mice (47) or mice immunised with NP-OVA. MOG-CFA and NP-HEL (31, 36). This will be discussed more when we describe the effect of immunosuppressive drugs on Tfr later on in this review. The modulation of the metabolism of GC B cells and Tfh could be another regulatory mechanism used by Tfr, leading to inhibition of production of IL-21 and IL-4 by Tfh and inhibition of class-switch recombination and antibody production by B cells (23). Tfr have been shown to produce TGF-β and IL-10 which is turn could lead to inhibition of B cells responses (47). It could be possible that Tfr produce granzyme B leading to B-cell and/or Tfh cytolysis (23, 47). Eventually, a direct mechanical disruption of Tfh and GC B cell has been hypothesised, but this has not been proven yet (47). Finally, Tfr regulates the interaction between Tfh and B cell during the GC reaction and limits the size of the GC reaction. They inhibit the production of high-affinity antibodies specific for self-antigens (48) and limits both self-reactive and non-specific responses (see **Figure 1**).

A distinct population of helper cells involved in B cell responses has been recently described by Rao et al. (49), as PD-1^hi^ CXCR5^−^ Bcl6^lo^and called T peripheral helper (Tph) cells. The original description of these cells was in a model of Rheumatoid Arthritis, but they have also been recently described as important in type 1 diabetes (50) and in the pathogenesis of lupus (51). It is currently not known whether Tfr can regulate this population, nor whether Tph are relevant to transplantation.

### Origin of Follicular Regulatory T Cells

Tfr have been found in spleen, lymph nodes and lymphoid tissues as well as in the lymphatic and blood circulations. Tfr cells were initially thought to arise from natural (thymus-derived) Tregs (12), that become induced upon TGFβ signaling in the periphery (14). Linterman et al. (12) found Tfr resembled Treg more closely than Tfh due to the elevated expression of many Treg associated genes; *FOXP3, Ctla4, Gitr, Klrg1*, and *Prdm1* as detailed above. However, Tfr also expressed high amounts of the *CXCR5, Pdcd1, Bcl6, CXCL13* (9) and *ICOS*, the typical Tfh genes. Tfr did not express receptors for the helper cytokines IL-21 or IL-4 or the costimulatory ligand CD40L. Furthermore Linterman et al., reported that 97% of Tfr cells express Helios, a transcription factor expressed by thymus-derived Treg cells. Thus, the origin of Tfr cannot be determined by genetic analysis alone.

To shed further light on the origin of Tfr, Linterman et al., transferred naïve cells (CD4^+^CD44^lo^CD25^-^) from mice expressing the 3A9 TCR transgene recognising a hen egg lysozyme peptide (HEL) into congenic mice. After being challenged with HEL, no donor originating Tfr could be identified with all Tfr deriving from recipient cells. Furthermore 6 days after selective ablation of all FOXP3^+^ Tregs using a diptheria toxin receptor inserted in the FOXP3 locus, Tfr were absent in diptheria treated mice, indicating that Tfr cannot form if FOXP3^+^ cells are absent, suggesting that Tfr development requires the presence of FOXP3^+^ Tregs.

Chung et al. (14), similarly sought to trace the origin of CXCR5^+^ Treg in mice. They found that CXCR5^+^FOXP3^+^ Tregs were essentially absent in the thymus compared to the spleen. To determine whether Bcl6^+^CXCR5^+^ Treg cells were generated from naïve CD4^+^ or natural Treg precursors in the periphery, they mixed CD45.1^+^ naïve CD4^+^ T cells (CD25^-^GITRCD44^lo^CD62L^hi^) and CD45.2^+^CXCR5^-^ Treg from FOXP3gfp mice, The T cells were injected into Tcrb-/- mice, which were deficient in alpha beta T-cell receptor and consequently had ~ 6% CD4^+^CD8^+^ of *wt* (52). This was followed by immunization with keyhole limpet hemocyanin (KLH) in complete Freund’s adjuvant (CFA). 98.2% of Bcl6^+^FOXP3^+^ cells in the recipient mice were CD45.2^+^, indicating that the origin of Bcl6^+^CXCR5^+^ Treg is from CXCR5^- ^Treg. Furthermore, they found that the majority of CXCR5^+^ FOXP3^+^ cells expressed Helios. While Chung et al. and Linterman et al., showed Tfr differentiate primarily from FOXP3^+^ Treg precursors, the former concluded that Bcl6^+^CXCR5^+^ Treg cells are absent in the thymus but induced in the periphery from CXCR5^-^FOXP3^+^ natural Tregs.

It may be that the differentiation of Tfr requires numerous stimulations. Thus, the thymus provides the microenvironment for Treg precursors to acquire CD31 (53) and Helios but then the subsequent differentiation of Tfr occurs by further stimulation in peripheral lymphoid tissue (15).

As a counter to Tfr deriving solely from FOXP3^+^ natural Tregs, Aloulou et al. (54), proposed they may also derive from FOXP3 negative precursors such as naive CD4^+^ T cells. They demonstrated that naive CD4^+^ T cells can become Tfr cells in murine models using an adjuvant that promotes peripheral Treg cell formation. This may occur in the context of a stimulus that promotes the conversion of CD4^+^ FOXP3^−^ cells into FOXP3^+^ Treg cells, specifically, one that enhances PD-L1 expression on antigen presenting cells. Whether these ‘induced’ Tfr cells emerge from Tfh cells that acquire FOXP3 expression, or from peripheral Treg cells that acquire the follicular fate through CXCR5 expression awaits further investigation. However, Tfh cells cannot be induced to switch on FOXP3 *in vitro* (13), once again suggesting that it is more likely that it is peripheral Treg cells that give rise to Tfr cells.

Again, the concept that Tfr can arise from FOXP3^-^ T cells has been countered by Maceiras et al. (55). They used congenic mice to investigate the precursors of Tfr cells following immunization in two distinct genetic backgrounds without the confounding issue of lymphopenia. They found that the adoptive transfer of thymic-derived FOXP3^+^ Treg in mice can differentiate into Tfr but FOXP3^–^ T cells only differentiate into Tfh. Additionally, they demonstrated that Tfh and Tfr pools are generated from distinct TCR repertoires, with Tfh cells expressing antigen-responsive TCRs to promote antibody responses, and Tfr cells expressing potentially autoreactive TCRs to suppress autoimmunity, strengthening the idea that Tfr and Tfh are derived from distinct populations. The proposed origin of Tfr in murine models is summarized in **Table 2**.

**Table 2:** Proposed cells of origin of Tfr in mouse models.

Reference	Conditions	Originating Cell	Definition of Tfr
***Linterman et al.,** Nat Med.* *(2011)* (12)	Selective ablation of FOXP3+ Tregs using diphtheria toxin meant no Tfr developed	FOXP3+ Tregs	CD4+CXCR5^high^PD-1^high^FOXP3+
***Chung et al.,** Nat Med.* *(2011)* (14)	Induced in the periphery from CXCR5-FOXP3+ natural Tregs	CXCR5- Treg	*CD4+Bcl6+CXCR5+FOXP3+*
***Aloulou et al.,** Nat Commun. (2016)* (54)	Incomplete Freund’s Adjuvant promotes conversion of CD4+FOXP3- cells into FOXP3+ Tregs	FOXP3- naive CD4+ T cells	*CD4+ CXCR5+PD-1+ FOXP3+*
***Maceiras et al.,** Nat Commun.* *(2017)* (55)	Adoptive transfer of thymic-derived FOXP3+ but not FOXP3- cells into congenic mice showed development of Tfr	Thymic-derived FOXP3+ Treg	*CD4+CXCR5+PD-1+FOXP3+*

As discussed above, early Tfr (eTfr), derive most probably from natural Tregs in the periphery, and, after expression of Bcl-6 and CXCR5, are attracted to the T cell zone of SLO, where they interact with activated dendritic cells (**Figure 1**). This priming step is required by the Tfr as the number of these cells were reduced in a model of immunised mice with 4-hydroxy-3-nitrophenylacetyl hapten–conjugated OVA (NP-OVA), where dendritic cells have been ablated (36). Within the T-zone eTfr can have two different fates. Following the interaction with follicular B cells, they can lose their Blimp-1 expression, upregulate Bcl-6, ICOS, and PD-1 and transfer to the T-B border in a CXCR5 dependent manner, becoming “intermediate Tfr” (iTfr) (15). Again, this step is crucial for full differentiation of Tfr, as Tfr were almost absent in draining lymph nodes of immunized mice that lack B cells (36). The molecular mechanisms associated with the interactions between follicular B cells and iTfr remains to be determined, however, there is some evidence that this step could be antigen-independent (36, 55–57). Conversely, eTfr can retain an immature phenotype, fail to express Bcl6, and access the circulation, becoming CD25^+^CXCR5^+^FOXP3^+^Bcl6^-^ blood Tfr (bTfr) (34, 36). Therefore, the presence of bTfr could be the footprint of a truncated GC formation regulation and the consequence of this could be an increase proliferation of Tfh and antibody production. In some auto-immune diseases the presence of bTfr in the blood correlated with the severity of disease (53, 58, 59). Although bTfr have been described in human (15), they have not been fully characterized and their function remain poorly understood. In particular, their precise role in transplantation remains to be elucidated.

The iTfr migrate then to the GC where they can become fully mature Tfr (mTfr) with very efficient suppressive capacities (30). It is at this stage that they lose the CD25 expression and upregulate Bcl-6, ICOS and PD-1 (60). In human, these cells are able, not only to inhibit Tfh activation (therefore decreasing their production of IL-4, IL-21) and suppress Tfh cell-GC B cell interactions leading to antibody production (61) but also decrease the activation of B cells through PD-1/PD-L1 interactions and the inhibitory function of CTLA4 molecules. Moreover, it has been demonstrated that they inhibit the capacity of class switching from IgM to IgG in mouse (23) and decrease IgA production by B cells in human (62), therefore inhibiting the selection of non-antigen-specific B cells (including those with self-reacting BCR) and limiting the number of B cells (indirect regulation). The regulation of B cells by Tfr is mechanistically complex and context-dependant as demonstrated very recently by Lopez-Ocasio Maria et al. (63). They showed with experiments *in vitro* that when the BCR was engaged, B cells were more resistant to suppression by Tfr, and this was dependent on a CD40-CD40L-associated mechanism.

The localization of these potential Tfr subtypes are not exclusive, and it has been demonstrated histologically that both iTfr and mTfr can be present in the Follicle and in the GC (60). It remains unknown if mTfr and iTfr could recirculate in the blood. However, it seems to be possible for bTfr to migrate back to Follicle and GCs (36). The Tfr regulation within GC seems to be happening in early GC formation stages (45), as Tfr seem to be less frequent in fully developed GCs (64) and might be inhibited by cytokines produced in GCs (65).

In summary, even if further work is needed in human, Tfr seem to derive from thymic Trefs. bTfr seems to be CD4^+^CD25^+^FOXP3^+^CXCR5^+^PD-1^low^ICOS^low^, whereas mTfr could be described as CD4^+^CD25^+^FOXP3^+^CXCR5^+^PD-1^+^ICOS^+^Bcl-6^+^. The exact role of these different subpopulations still needs to be investigated, particularly in the context of transplantation.

## Role of Tfr in Human Diseases

Tfr have been described in responses to influenzae vaccination and chronic infections associated with hepatitis C virus (HCV), human immunodeficiency virus (HIV) and hepatitis B virus (HBV) (46, 66–68) and they are particularly relevant in the settings of autoimmune (AI) diseases. Tregs are at the core of the physiopathology of autoimmune diseases as their role is to regulate the responses to self-antigen as demonstrated by an association between autoimmune conditions and defects in Treg function (37). Tfr have been described and characterized in several autoimmune diseases (53, 58, 59, 69). They represent a critical peripheral tolerance mechanism, to prevent GC derived auto-immunity. Patients suffering from auto-immune diseases may have an unbalance of Tfr between blood and LN in favour of blood naïve Tfr, leading to a non-specialized response in the LN (59). In this review, we will focus on the role of Tfr and their impact on the immune responses in the setting of bone marrow and solid-organ transplantation.

### Relevance of Tfr in Bone Marrow Transplantation

Graft *versus* host disease (GvHD) is a significant complication of allogeneic hematopoietic stem cell transplantation, whereby transplanted donor cells recognise recipient antigens as foreign. This may be acute (aGvHD) or chronic (cGvHD). In cGvHD, alloreactive Tfh cells and germinal center (GC) B cells have a crucial role in GC reactions to produce pathogenic antibodies, as evidenced by the reduction in severity of cGvHD in mouse models when these antibodies are inhibited (70). Although Tfr can inhibit GC reactions by acting as negative regulators of B cell function (71), Treg numbers are reduced in patient samples of cGvHD (72) likely contributing to cGvHD pathogenesis.

McDonald-Hyman et al. (73) found mice with cGvHD had significantly fewer Tfr in line with patient data, suggesting that a loss of regulation by Tfr associates with cGvHD. However daily therapeutic interleukin-2 complexed with the JES6-1 clone of anti-IL-2 antibody (IL-2/mAb) increased Tfr numbers, due to the fact they preferentially bind to CD25^hi^ cells, while Tfh numbers were consequently reduced. Markers of cGvHD were also reduced as assessed by tissue pathology scores and pulmonary function tests. This effect was not seen in aGvHD since treatment with IL-2/mAb complexes led to an expansion of total Tregs and CD8^+^ Tconv likely counterbalancing Treg expansion. They did not examine Tfh or Tfr subsets in this context.

The same authors also tested *wt* Treg infusions in cGvHD, which increased Tfr, while Tfh and GC B-cell frequency, GC size, and tissue pathology scores were significantly reduced in these mice. Thus this implies boosting Tfr can ameliorate cGvHD. They found that these events were CXCR5 dependent since CXCR5KO Tregs given during cGvHD once GC had formed had no effect on lung function, Tfh or GC B cells but did have improve lung function if given prophylactically. The lack of any effect correlated with the Tfr numbers that was not increased but was to a similar level to the one observed in mice with cGvHD. This suggests the importance of targeting Tregs homing to formed GC. Furthermore, the miR‐17–92 cluster has been found to facilitate Tfh‐cell differentiation and impair Tfr/Tfh balance, thus accelerating the development of cGvHD in mice (74).

Comparatively, Kamihara et al. (75) performed functional assays and flow cytometry on cryopreserved human PBMC of healthy donors and those undergoing allogeneic stem cell transplantation and those with active GvHD on IL2 therapy. Numbers of Tfr were significantly reduced in those who had undergone allo-SCT compared to healthy donors (median 0.08 vs 0.34% of CD4^+^ T cells respectively). Patients with active cGvHD also had significantly lower Tfr cell frequency compared to matched patients with none or resolved cGvHD. *In vivo* administration of low dose IL-2 therapy for one week led a selective expansion of Tfr which remained stable during the 12 weeks of therapy. The Tfr had increased expression of CD25, FOXP3, CTLA-4, ICOS, Helios, Ki67 Bcl6, and p-STAT5. In contrast, activated ICOS^+^PD-1^+^ circulating Tfh were suppressed during IL-2 therapy. The selective activation of circulating Tfr and suppression of circulating Tfh provide a mechanism whereby low dose IL-2 therapy can promote both B and T cell tolerance in patients with cGvHD. In summary, these studies suggest a reduction of Tfr correlates with active or chronic GvHD and by boosting numbers of Tfr, either with cell infusion or IL-2, this may prove an effective therapeutic strategy.

### Relevance of Tfr in Solid Organ Transplantation

#### Tfr in Transplantation and Alloantibody Responses

The role of Tfh in transplantation and alloantibody formation has been extensively studied over the last decade (76–78). Some human observational studies have described an increase in bTfh cell numbers in patients with transplant rejection (78, 79), and a reduced proportion of bTfh cells in patients with operational tolerance (80). Tfh cells have been shown in biopsies of patients with acute kidney rejection (77) and in ectopic lymphoid structures in kidney biopsies of acute T cell rejection (81). However, the role of Tfr in the alloimmune context needs to be explored. Some have postulated that Tfr prevent antibody responses in the context of low levels of antigen (36) and/or when low-affinity BCR are produced after somatic hypermutation (12). These situations do not fit with the transplant setting where the antigens are persistent.

#### Tfr in Acute and Chronic Rejection in Solid Organ Transplantation

Although the exact role of Tfr in transplantation and in antibody production needs further investigation, analysis of phenotype and/or frequency and/or function of these cells might be helpful for diagnostic purpose. Extensive work has been done to link Tfh with rejection (82, 83), or exploring Tfh/Tfr ratio in the context of autoimmune diseases (59). The published data evaluating Tfr in solid organ transplantation other than kidney is scarce. One study in a mouse model of lung transplantation (84) has demonstrated that bronchus-associated lymphoid tissue-resident FOXP3^+^ T lymphocytes expressing CXCR5 were responsible for the prevention of antibody-mediated rejection. **Table 3** summarize the studies of Tfr in kidney transplant patients. The definition of Tfr varies and the markers selected to characterize the Tfr do not allow to distinguish between the different subtypes of Tfr (bTfr vs iTfr or eTfr). However, there seems to be a trend toward lower levels of bTfr and chronic rejection (79, 86) and a potential correlation between low levels (79, 86) of bTfr and reduced kidney function as demonstrated by low estimated glomerular filtration rate (eGFR) (79). However, the number of bTfr in the transplanted patients was not compared to a group of patients with kidney dysfunction. Moreover, the definition of CAD used in these studies was not uniform or not clearly stated. Therefore, it is difficult to know if the decrease in bTfr numbers was secondary to the degree of uraemia (87). As yet Tfr in patients with end-stage renal disease (ESRD) or on haemodialysis has not been assessed. These results could seem inconstant with the findings of high bTfr in the auto-immune settings described above, however there are no definite explanation for these apparent inconstancies. Only two studies managed to collect some lymphoid structures from transplanted patients: Tfr were present in LN from a patient who had a kidney transplantation (62) but rare in kidney tertiary lymphoid organs in patients with either chronic or acute rejection (85). In conclusion, the current evidence is trending toward a decrease of bTfr in patients with CAD, however this needs to be confirmed with bigger numbers of patients and a more consensual definition of CAD.

**Table 3:** Studies focusing on T-follicular regulatory cells in human and solid-organ transplantation.

Reference	Conditions	Loc	Definition	Major findings	Comments
*Wallin EF et al, Blood (2014)* (62)	- 5 KTR ttt with rituximab - 21 controls	*Lymph node*	CD4+ CXCR5+ FOXP3+ CD127- CD57+	- Tfr are present in LN of KTR - Rituximab had no impact on Tfr cells - Tfr cells reduce IgA production by B cells *in vitro*	
*Xu X et al, Immunol Invest (2016)* (85)	- 29 CAR - 5 hyperacute rejection - 12 acute allograft rejection	*Kidney*	CXCR5+ FOXP3+	- Tfr were rarely present in kidney tertiary lymphoid structures	Definition of CAR, acute rejection not clear
*Chen W et al, Scientific reports (2017)* (86)	- 88 KTR with chronic allograft dysfunction (CAD) incl.40 with biopsy proven ABMR - 30 controls	*Blood*	CD4+ CXCR5+ ICOS+ FOXP3+ CD127-	- ABMR: lower numbers of bTfr and kidney Tfr compared to non AMBR CAD+ - Tfr from ABMR display normal inhibitory function - Sirolimus decrease ratio of Tfr - Tfr inhibit B cell proliferation and differentiation in KTR - Tfr regulation of B cell is dependent on CTLA4	No definition of CAD
		*Kidney*	CD4+ CXCR5+ FOXP3+		
*Yan L et al, BMC Immunology (2019)* (79)	- 34 CAD incl. 21 with biopsy (11 ABMR, 2 TCMR, 9 no rejection) - 33 controls	*Blood*	CD4+ CXCR5+ FOXP3+	- Decreased frequency of bTfr and increased bTfh : Tfr ratio in CAD group - Increased serum CXCL13 and decreased serum TGF-β in CAD - bTfh:bTfr independent risk factor for low GFR and CAD	CAD = eGFR < 60 ml/min/1.73m^2^ after 3 months post KT
*Niu Q et al, Frontiers Immunol (2020)* (61)	- 211 KTR 5-7 years after Tx, inc 24% with background of rejection - 30 controls	*Blood*	CD3+ CD4+ CXCR5+ FOXP3+	- Decreased cTfr to cTfh in transplanted patient compared to controls - No association between anti-HLA antibodies or DSA and cTfr ot Tfh	

KTR, kidney transplant recipients; Tfr, T-follicular regulatory cell; cTfr, circulating Tfr; bTfr, blood Tfr; LN, lymph nodes; IgA, immunoglobulin A; GC, germinal centers; CAD, chronic allograft dysfunction; CAMR, chronic antibody mediated rejection; ABMR, antibody-mediated rejection; TCMR, T-cell mediated rejection; ttt, treated; CAR, chronic allograft rejection; Tx, transplantation; HLA, human leucocyte antigen; DSA, donor-specific antibodies.

#### Tertiary Lymphoid Organs

Tertiary lymphoid organs (TLOs) are ectopic lymphoid aggregates frequently observed in tissues affected by non-resolving inflammation as a result of infection, autoimmunity, cancer, and allograft rejection (88–91). They vary from tight clusters of T and B cells to highly ordered structures resembling the cellular composition of lymphoid follicles typically associated with secondary lymphoid organs (SLOs), such spleen and LN (90). The process whereby inflammatory cells infiltrate chronically rejected grafts and are progressively organised into structures has been termed lymphoid neogenesis (92). Although TLOs within tissues show varying degrees of organization, they frequently demonstrate segregated T and B cell zones, follicular dendritic cell networks, a supporting stromal reticulum, and high endothelial venules. In this respect, they mimic the activities of germinal centers and contribute to the local control of adaptive immune responses. However, unlike SLOs but akin to mucosa-associated lymphoid tissue, TLOs do not have afferent lymph vessels and are not encapsulated, suggesting they are directly exposed to local antigens or cytokines (93). Studies in various disease settings have described how these structures can contribute to either beneficial or harmful outcomes. In the context of transplantation and DSA production whether lymphoid neogenesis is harmful, beneficial, or simply a bystander occurrence remains to be fully elucidated.

Several groups have reported that TLOs can amplify anti-graft immunity and accelerate tissue destruction. In murine studies of heart and skin transplantation, TLO formation has been associated with rejection (89). Infiltrating lymphocytes were found to be composed of both B cells and follicular-helper like CD4+ T cells in rat aortic allografts, and to be associated with antibody production independent of SLOs, suggesting local antibody production (85). This group went on to find ectopic GCs in all explanted human cardiac (n=5) and renal allografts (n=24) undergoing chronic rejection but not control organs (88). Histological examination revealed B cells near CD23^+^ follicular DC surrounded by CD3^+^ T cells, although further phenotyping to identify Tfr was not done.

Other studies suggest that the presence of Tregs in TLO might promote graft tolerance, thereby slowing down the kinetics of chronic rejection (92, 94–98). Xu et al. (85) measured the distribution of TLOs and the expression of FOXP3 and CXCR5 in explanted human renal allografts with chronic rejection. FOXP3^+^ Tregs were detected in 10/29 chronically rejected grafts and 1/12 acutely rejected grafts and this did not correlate with lymphoid neogenesis or prolonged graft functioning. CXCR5^+^ FOXP3^+^ Tfr cells were rare in both chronically and acutely rejected grafts with TLO vs. those without. CXCR5^+^ FOXP3^+^ cells were present in 7/29 chronically rejected grafts but none of the acutely rejected grafts, implying Th17 but not Tfh could be involved in lymphoid neogenesis.

In summary, the possibilities remain that 1) lymphoid neogenesis is simply an epiphenomenon related to graft duration as proposed by Thaunat (92), 2) that Treg numbers are diminished in rejected organs in TLO, or 3) that there is another regulatory cell that aids TLO-mediated tolerance.

#### Effect of Immunosuppression

Regardless of the type of organ transplanted, recipients require some degree of immunosuppression to prevent allograft rejection; this is usually in the form of induction and then subsequent lifelong maintenance immunosuppression. Many of the immunosuppressive agents are T cell targeted and result in disruption of T cell homeostasis. A summary of studies focusing on the effect of immunosuppressive agents on human Tfr is presented in **Table 4**.

**Table 4:** Summary of studies focusing on the effect of immunosuppressive agents on human Tfr.

Reference	Conditions	Sample	Definition	Major findings
*Wallin EF et al,* *OBM Transplant. (2019)*	Alemutuzumab induction for 19 SPK and 23 KTR vs 18 basiliximab treated KTR	Blood	CD4+ CXCR5+ FOXP3+ CD127lo	Tfr and Tfr : Tfh significantly lower in alemtuzumab treated patient up to 24 months post-transplant Trend toward lower Tfr in those developing *de novo* DSA
*Wallin EF et al,* *Front Immunol. (2018)* (99)	16 live donor KTR 1 week pre-treated with tacrolimus vs 45 deceased donor SPK or KTR	Blood and lymph node	CD4+ CXCR5+ FOXP3+ CD127lo	Decreased bTfh and lymph node Tfh. Trend towards fewer Tfr Co-culture of memory B cells and Tfh with tacrolimus showed lower plasmablast differentiation and antibody production
*Chen W et al, Scientific reports (2017)* (86)	30 controls	Blood	CD4+ CXCR5+ FOXP3+ CD127− ICOS+	In vitro tacrolimus increased Tfh1, decreased Tfh2 and Tfh17, no change in Tfr In vitro rapamycin reduced ratio of Tfr, no effect on Tfh1, Tfh2, and Tfh17 cells
	88 KTR with CAD incl.40 with biopsy proven ABMR			Overexpression of CTLA4 increased Tfr proportion and associated with less B cell proliferation
*Niu Q et al, Expert Rev* *Clin Immunol. 2019* (100)	KTR on Tac, MMF and steroids	Blood	CD3+ CD4+ CXCR5+ FOXP3+	Lower numbers of bTfr associated with anti-HLA antibodies and worse renal function
*Niu Q et al,* *Front Immunol. (2020)* (61)	211 KTR 5-7 years after Tx, inc 24% with background of rejection 30 controls	Blood	CD3+ CD4+ CXCR5+ FOXP3+	Lower bTfr, no difference in bTfh, thus decreased bTfr : Tfh in KTR vs HC Previous rejection had reduced Tfh and Tfr – Tfr : Tfh same No association between anti-HLA antibodies or DSA and bTfr to Tfh Alemutuzumab and MP treated patients had significantly lower bTfr and bTfh

SPK, simultaneous pancreas kidney transplant; KTR, kidney transplant recipients; Tfr, T-follicular regulatory cell; bTfr, blood Tfr; CAD, chronic allograft dysfunction; ABMR, antibody-mediated rejection; Tx, transplantation; HLA, human leucocyte antigen; DSA, donor-specific antibodies; MP, methylprednisolone; Tac, tacrolimus; MMF, mycophenolate mofetil; HC, healthy controls.

##### Early Transplant Immunosuppression

Induction T cell depleting agents include anti-thymocyte globulin (ATG) and alemtuzumab which is a monoclonal antibody against CD52 expressed by most lymphocytes. Comparatively, another induction agent basiliximab, is a non–T cell depleting monoclonal antibody specific for the IL-2Rα receptor (CD25). Blockade of CD25 is designed to prevent T cells activation, in part by blocking the effect of autocrine IL-2 production and also to reduce T cell activation of B cells (101). Whether it affects Tfh and Tfr equally is unclear.

Lymphopenia-induced proliferation after depletion initiates repopulation of CD4^+^ T cells with a phenotype skewed toward effector memory pool and a significant decrease in the naïve pool (102). These cells have a lower threshold for activation, can circulate to the graft and are less dependent on costimulation for activation (56). Thus, a predominance of memory T cells could contribute to graft injury and rejection. Conversely, the frequency and memory differentiation of CD4^+^ T cells post-basiliximab induction remains unchanged (103).

In an ATG treated group of renal transplant patients, Macedo et al. (104) found that the absolute numbers of circulating Tfh (CD45RO^+^CXCR5^+^CD4^+^CD3^+^) were significantly decreased at all time points up to 360 days post induction. The percentage of Tregs was overall decreased in the ATG group, although they did not evaluate the Tfr numbers. Furthermore, the Tfh repopulation following ATG was found to be skewed toward Th1 polarization and effector memory. There was also a correlation of higher Th1 polarized cTfh cells relative to Tregs numbers in those that developed DSA. This suggests that rising Tfh numbers post-transplant may associate with DSA occurrence in the context of ATG induction.

Wallin et al. (105) compared induction with alemtuzumab in simultaneous pancreas-kidney (SPK) vs. renal transplant patients receiving basiliximab. CXCR5^+^IL-7R^lo^FOXP3^+^CD4^+^ bTfr cells remained significantly lower in alemtuzumab treated patients, both in proportion of total CD4 population and absolute cell count (compared to basiliximab patients at almost all time points up to 24 months post-transplant) despite being significantly higher in this group prior to transplant. There was also a trend toward a lower proportion of CXCR5^+^IL-7R^lo^FOXP3^+^CD4^+^ Tfr cells in patients developing *de novo *DSA compared to those who did not, but this was not statistically significant owing to low patient numbers developing *de novo *DSA.

Overall, the ratio of circulating Tfr : Tfh between treatment groups was significantly lower in all alemtuzumab patients compared to basiliximab treated patients up to 24 months post-transplant, reflecting the persistent low levels of Tfr cells in alemtuzumab treated patients despite a recovering bTfh population. The fact that both alemtuzumab use and *de novo* DSAs associates with a pattern of low bTfr:cTfh is one putative explanation of why alemtuzumab patients develop higher rates of *de novo* DSA post-transplant (106), but necessitates further elucidation to establish causality.

Glucocorticoids are frequently used at least in the early stages of post-transplant immunosuppression or during rejection. They act *via* inhibition of cytokines such as IL-1, TNF alpha, IFNγ and IL-6. A study by Wen et al. (107) found a correlation between 3 months of glucocorticoid use in thirteen previously untreated myasthenic patients and an increase in circulating Tregs and Tfr cells with a reduction in circulating Tfh. They inferred that treatment with steroids can attenuate the symptoms of myasthenia gravis by restoration of the imbalances between circulating Treg, Tfr, and Tfh and maintaining immune homeostasis. This suggests that steroids may exert a similar effect in transplant recipients by promoting a positive ratio of Tfr : Tfh. This was also seen in autoimmune treatment with 5mg prednisolone causing an increase in Tfr compared to pre-treatment.

In the context of renal transplant rejection Seissler et al. (108), found methylprednisolone (125-250mg) for 3 days did not affect the percentage of Treg numbers (CD4^+^FOXP3^+^CD127^lo+/−^) within the total CD4^+^ T cell population. However, the ratio of different Treg subsets changed such that DR^+^CD45RA^−^ Tregs increased significantly, while the naïve DR^−^CD45RA^+^ Tregs decreased significantly. Moreover, they observed a disproportionally strong expansion of the DR^high+^CD45RA^−^Tregs which have been shown to have maximal suppressive properties (108). However, this proportional increase in DR^high+^CD45RA^−^Tregs was not sustained beyond 3 days and may be confounded by the effects of increasing other immunosuppressive doses.

##### Longer-Term Immunosuppression

For maintenance immunosuppression, post-transplant patients are commonly on calcineurin inhibitors which remain a cornerstone of immunosuppression regimes. Tacrolimus (Tac) and cyclosporin (Csa) are often used and exhibit their action *via* blockade of the dephosphorylation of the nuclear factor in activated T cells (NFAT) (109). This prevents translocation into the DNA promoter region in the nucleus, thereby selectively suppressing the cytokine gene transcription for IL-2 (110), TNF-a, IL-3 and IL-4 (111). This affects T cell proliferation and activation (112). Vaeth et al. (20), found that Tfr are highly dependent on NFAT signaling indicating that CNI could plausibly impair the function of these subsets (113).

Wallin et al. (99), compared the effect of tacrolimus on paired blood and lymph node samples from transplant recipients. Living-donor kidney transplant recipients were treated with tacrolimus for a week prior to transplantation while the deceased-donor recipients received no pre-transplantation tacrolimus. One week of treatment reduced the frequency of both circulating and lymph node Tfh cells in the transplant recipients. At the same time, Treg remained the same in both tacrolimus treated and untreated recipients. There was a trend to toward fewer Tfr (CXCR5^+^IL-7R^lo^FOXP3^+^CD4^+^ cells) numbers and as a proportion of total CD4^+^. Comparatively, 11 days of tacrolimus administered to Tfh in co-culture with memory B cells lead to lower PD-1 expression, plasmablast differentiation and antibody production compared to control treated cells.

Further *in vitro* evidence indicates Tac administration to Tfh-B co-culture prevented plasmablasts and IgG formation in cells from renal transplant patients, suggesting it is Tac targeting of Tfh that prevents DSA formation (114). Similarly, in healthy volunteers, Chen et al. found that Tfr cell proportions were unaffected by treatment with CNI but the Tfh1 percentage increased while the IL-21 producing Tfh2 and Tfh17 cells decreased (86). The results from these studies may be influenced by the use of healthy volunteers in Chen’s study vs. transplant recipients. Thus, CNIs are likely to have a dominant effect on Tfh rather than Tfr. This may be explained by higher expression of NFAT in Tfh cells than in other CD4 subsets (20). Alternatively, suppression of Tfr cells, like Tregs, may require higher doses or longer duration of CNI treatment than required for suppression of Tfh cells.

Antiproliferative agents, such as mycophenolate mofetil (MMF), are another commonly used immunosuppressive drug. This is a pro-drug which undergoes hydrolyzation by gut esterases to give the active mycophenolic acid. This acts by inhibiting inosine monophosphate dehydrogenase, which is crucial for purine synthesis in T and B cells. However, there are conflicting reports of MMF promoting induction of Tregs from Tconvs (115), while others suggest a dose dependent reduction in Treg viability and proliferative capacity (116). The specific effect on Tfh and Tfr has not been examined in the literature.

A less commonly used agent is sirolimus, a mammalian target of rapamycin inhibitor (mTOR). The mTOR signaling pathway plays a crucial role in dictating T cell fate through the interaction and balance of two mTOR containing complexes, mTORC1 and mTORC2. Xu et al. (117) found mTORC1 was expressed at high levels in mouse Tfr cells. By deleting the essential components of mTORC1 and mTORC2 they demonstrated that mTORC 1 but not mTORC2 was essential for Tfr differentiation, which was *via* the p-STAT3-TCF-1-Bcl-6 pathway. Essig et al. also found mTOR inhibitors suppressing PI3K-mTOR signaling inhibits the conversion of Treg to Tfr cells (118). Xu et al. showed that Tfr differentiated from mouse Tregs in the presence of rapamycin had lower expression of CXCR5, GITR and CTLA-4 compared to vehicle treated precursors. Tfr derived in the presence of rapamycin also had reduced suppressive function as indicated by an increased proportion and total number of GC B cells in spleens compared with the spleens of mice that had received *wt* Tfh and vehicle-treated Tfr cells. Essig et al. (118) used Roquin, an RNA-binding protein, to inhibit the PI3K-mTOR pathway at several levels, noting that differentiation toward Th17 and Tfh (PD1^int^CXCR5^int^CD4^+^) was inhibited as well as Treg to Tfr (CXCR5^hi^ PD-1^hi^ FOXP3 CD4^+^).

Chen et al. (86) found 48-h *in vitro* culture with rapamycin could reduce the ratio of CD4^+^CXCR5^+^ICOS^+^FOXP3^+^CD127^−^ Tfr cells in healthy volunteers but had no effect on Tfh1, Tfh2 and Tfh17 cells. Thus, mTOR inhibitors may adversely affect Tfr cell function, skewing the balance toward Tfh cells. This is supported by mouse models suggesting that the use of mTOR inhibitor rapamycin after alemtuzumab induction increased the proportion of Tfh cells while significantly reduced the number of Tregs 2 weeks post cardiac transplantation and was associated with an increase in DSAs (119).

Another key mediator of Treg function is CTLA-4, which contributes to the suppressive function of Tfr by downregulating the expression of CD80/86 on antigen presenting cells and consequently reducing CD28 engagement (120). Treg-specific deletion of CTLA-4 results in a massive increase in antibody production, pointing to a crucial role for CTLA-4 on Treg cells in limiting B cell responses (120). Wing et al. subsequently (71) showed that murine CTLA-4 deficient Tfr (CXCR5^+^ Bcl6^+^ FOXP3^+^) either from CTLA-4 KO mice or using anti-CTLA Fab were less able to reduce the expression of CD80 and CD86 on B cells. They also were less able to prevent effector T cell proliferation when purified B cells were used as stimulators. Thus, CTLA-4-deficient Tregs and Tfr exhibited significantly reduced, but not a total loss of, suppressive function *in vitro*.

Sage et al. (47) demonstrated murine Tfr (CD4^+^CXCR5^+^ICOS^+^FOXP3^+^CD19^−^) had very high expression of CTLA-4 compared to Tfh CD4^+^CXCR5^+^ICOS^+^FOXP3^−^CD19^−^. In their mouse model where CTLA-4 is conditionally deleted on FOXP3^+^Tregs upon tamoxifen administration, they found substantial increases in total Treg and Tfr numbers, ICOS expression and an increase in Tfr : Tfh after tamoxifen. However, these Tfr showed diminished suppressive capacity of B cell function in suppression assays both *in vitro* and *in vivo*, as indicated by a substantial increase in antigen specific IgG. Chen et al. (86) used a lentivirus to overexpress CTLA-4 in Tregs from renal patients with CAD finding an increased proportion of human Tfr (CD4^+^CXCR5^+^ICOS^+^FOXP3^+^CD127^−^) and significantly less B cell proliferation. ELISA results showed Tfr inhibited IgG and IgA production from plasma cells. Conversely, there was increased proliferation and differentiation to plasma cells in the Tfr deleted group. This suggests that selectively increasing Tfr *via* CTLA-4 may be a good strategy to treat AMR by preventing B cell proliferation and differentiation. 

Belatacept is a CTLA-IgG fusion protein which binds to the CD80 and CD86 molecules on antigen presenting cells preventing T cell co-stimulation and promoting expression of indoleamine 2,3-dioxygenase. Oh et al. (114) showed CTLA-Ig in addition to rapamycin increased Tfh (ICOS^+^ PD-1^+^ CD4^+^) cells but not Tregs (CD4^+^ CD25^+^ FOXP3^+^) in a murine cardiac transplant model with alemtuzumab induction. In clinical transplantation, the impact of belatacept on Tregs has been difficult to assess because it is administered in combination with blocking antibodies targeting IL-2Rα and cyclosporin (121). Despite this, it is currently widely accepted that the use of high doses of CTLA4 Ig is detrimental to Treg survival, whereas low doses of CTLA4 Ig, unable to saturate CD80 and CD86, may favour Treg expansion to some extent in the long term (122). Kim et al. (123) in a primate renal transplant model found CXCR5^+^Bcl6^+^PD-1^hi^CD4^+^ T cells were greatly reduced in lymph nodes of costimulatory blockade treated (belatacept or anti-CD40 mAb) animals with AMR that had lower levels of DSA compared to primates with AMR receiving the control immunosuppression regimen.

While these studies involving immunosuppression are confounded by small patient numbers, differences in HLA typing and variation in immunosuppression regimes, there is a consistent finding that a positive ratio of Tfr : Tfh correlates with immune regulation and a trend toward less DSA formation. Niu et al. (100) observed that kidney transplant recipients on immunosuppressive therapy with tacrolimus (Tac), mycophenolate mofetil (MMF), and steroids, with anti-HLA antibodies including DSA had lower numbers of circulating Tfr (CD3^+^ CD4^+^ CXCR5^+^ FOXP3^+^) cells than patients without anti-HLA antibodies. Inverse correlations between the kidney function parameters (serum creatinine level), and number of circulating Tfr cells and number of Helios+ circulating Tfr cells were found, indicating that the reduction of number of bTfr cells might reflect the lack of regulation of active B cell immunit directed against the allograft in kidney transplant recipients.

Niu et al. (61), evaluated Tfr in the clinical context of long term multiple agent immunosuppression in 211 patients with a functioning renal transplant over 5 years post transplantation. They found absolute numbers of Tfr (total CD3^+^CD4^+^CXCR5^+^FOXP3^−^) including subsets that were PD1+ and Helios+, were lower in transplant recipients compared to healthy controls. There was no difference in total Tfh (CD3^+^CD4^+^CXCR5^+^FOXP3^−^), meaning consequently there was also a decrease in the Tfr : Tfh. Although different induction agents were used according to clinical indication, the majority being anti-CD25 mAb, most patients had similar maintenance regimes; tacrolimus, MMF and tapering prednisolone to 0 at 4–5 months. They compared 162 patients with no history of rejection vs 49 with rejection (5 presumed and 44 with biopsy proven), noting that those with previous rejection had lower numbers of Tfr and Tfh such that the Tfr : Tfh remained the same as those without rejection. However, blood samples were taken at a median of 4.9 years post rejection episode thus cell numbers may be as a consequence of specific anti-rejection treatment, enhanced overall immunosuppression or reflecting a pattern that predated rejection. Of the anti-rejection treatments, Alemtuzumab in combination with methylprednisolone was the only one associated with significantly lower numbers of both bTfr and bTfh cells, including their subsets.

One of the limitations of this study was the use of healthy controls as a comparison without CKD. Although the transplant recipients had functioning grafts, their eGFR was <40ml/min and they demonstrated that lower eGFR correlated with lower Tfr. Thus, a comparison to CKD patients with a GFR may have controlled for the confounding effect of uraemia rather than contrasting with healthy controls.

Overall, there appears to be a reduction of Tfr associated with most immunosuppression regimens. However, elucidating each agent’s effect is substantially confounded by the use of other drugs and frequent clinician adjustment in the context of rejection, infection etc. There has also been a trend in some studies (100) of a positive Tfr : Tfh associating with less DSA formation. However, Niu et al. (61), found patients with anti-HLA antibodies or DSA at 5–7 years post-transplant or any form of rejection had similar bTfr and bTfh cell numbers as those without antibodies or rejection. This is in opposition to Macedo et al. (104), who demonstrated lower Tfh. Similarly, a longitudinal study by Cano-Romero et al. (124), indicated that cTfh (CD4^+^CXCR5^+^PD1^hi^CCR7^lo^) expanded significantly more after transplantation in patients who developed *de novo* anti‐HLA antibodies than in patients who remained unsensitised. Thus, a contemporaneous longitudinal study of both Tfr and Tfh numbers and their functional assessment may indicate which patterns associate with immunosuppression changes, rejection and *de novo* DSA development.

## Cell Therapy Using Tfr

### Tregs Therapy

Over the last decade, clinical trials using autologous Tregs have grown exponentially, with not less than 52 current Treg clinical trials registered in January 2020 on ClinicalTrials.gov, two of which have involved our group. In solid organ transplantation, two trials in liver transplantation have been published (125, 126). One was suspended because of rejection in three patients even if seven were successfully weaned of immunosuppression (125), though there were some concerns about the potential presence of antigen-specific effector cells in the cell product used in this study. The ThRIL study (126) demonstrated that Treg infusion in liver transplant recipients was safe. It was easier to achieve the aim of delivering 4.5 millions of Tregs per kilogram after expansion if the Tregs were isolated 6–12 months after liver transplantation, in comparison to trying to identify patients to treat while they were awaiting their transplant. Treg infusion resulted in a transient increase of the pool of circulating Tregs. Patients who received the full 4.5 million/kg Tregs product displayed a decrease in T cell responses against donor cells (assessed by the upregulation of CD154 on memory CD8^+^ Tcells).

The One study (127) presented the results of seven trials using different types of regulatory cell products, used in place of induction treatment for kidney transplantation. The results were not individualized for each type of cell products but demonstrated that the use of regulatory cell products is safe and could even lead to a decrease to post-transplant viral infections. Patients receiving Tregs products displayed more stable Treg-specific demethylated region compared to those receiving standard of care. However, those studies have used autologous polyclonally expanded Tregs and the proportion of Tfr in these products was not investigated.

These have led to the development of an ongoing Phase IIb study in kidney recipients (TWO Study, https://doi.org/10.1186/ISRCTN11038572) and to a Phase IV study in liver recipients, with the association of low dose IL-2 infusions (LITE study: NCT02949492). The latter, which tested the efficacy of low dose IL-2 infusions in liver transplant recipient as potential therapy to increase autologous Tregs *in vivo* was stopped prematurely, as some patients developed rejection on protocol biopsies.

### Access to Tfr and Cell-Engineering

The biology and role of different subsets of Tfr remains to be further elucidated in humans. However, bTfr seem to be good candidates for autologous therapy. First, they are accessible by leukapheresis (in contrast to their lymph nodes counterparts) and display a memory-type phenotype (36). Moreover, they are easily identifiable and could potentially be sorted from fresh blood as they could be defined as CD4^+^CD25^+^ CXCR5^+^ cells. However, their suppressive capacity, in comparison to mTfr has not been tested and it remains unknown whether they will be recruited into GC and become fully mature. Moreover, the frequency of bTfr in human blood and whether they can be expanded *in vitro* are both unclear.

As Tfr are derived from Tregs, it could be possible to use general Tregs, and modify them so that they become Tfr. Kim et al. (128), used retroviral transduction of the CXCR5 gene in FOXP3^+^ Tregs and demonstrated stable expression of functional CXCR5 on transduced-Tregs. CXCR5-transduced Tregs maintained a Treg signature and suppressive activity *in vivo* after adoptive transfer in mice. Moreover, using a transwell culture system, Kim et al. demonstrated that they migrated efficiently down a CXCL13 gradient and suppressed antibody production by B cells.

The field of tailored cell therapy has advanced enormously in recent years thanks to new engineering techniques including CRISPR-Cas9 (129) and chimeric antigen receptor (CAR) technologies (130, 131). The latter involves synthetic fusion proteins which typically combine an extracellular antibody-derived antigen targeting moiety and an intracellular TCR complex-derived signaling domain (or domains). The resultant protein is consequently able to bind designated target antigens, in an MHC-independent manner, and translate this engagement into activation of customized T cell signaling cascades [reviewed by us (132)]. The first Phase 1/2 human clinical trial using CAR-Tregs (in a setting other than cancer) will launch in 2020 (STEADFAST study, Sangamo Therapeutics) and will test CAR-Tregs in prevention of immune-mediated rejection following HLA-A2 mismatched kidney transplantation. Using a CXCR5-CAR on Tregs could help them to migrate to the lymph node and potentially inhibit antibody responses. Fine tuning the expression of CXCR5 seems to be more appropriate and as the Bcl-6 dependant CXCR5 expression is a dynamic process, using CRISPR-Cas9 technology to incorporate Bcl-6 to Tregs genome might be another track to explore.

Although promising and full of potential, cell-engineering might not be appropriate for Tfr production, as these cells cannot be defined by the presence of one parameter. More than producing Tfr through an engineering process, expanding Tfr from isolated Tregs might be another tempting option.

### Applicability of Treg Expansions Protocols to Tfr: IL-2/Rapamycin

**Figure 2:**
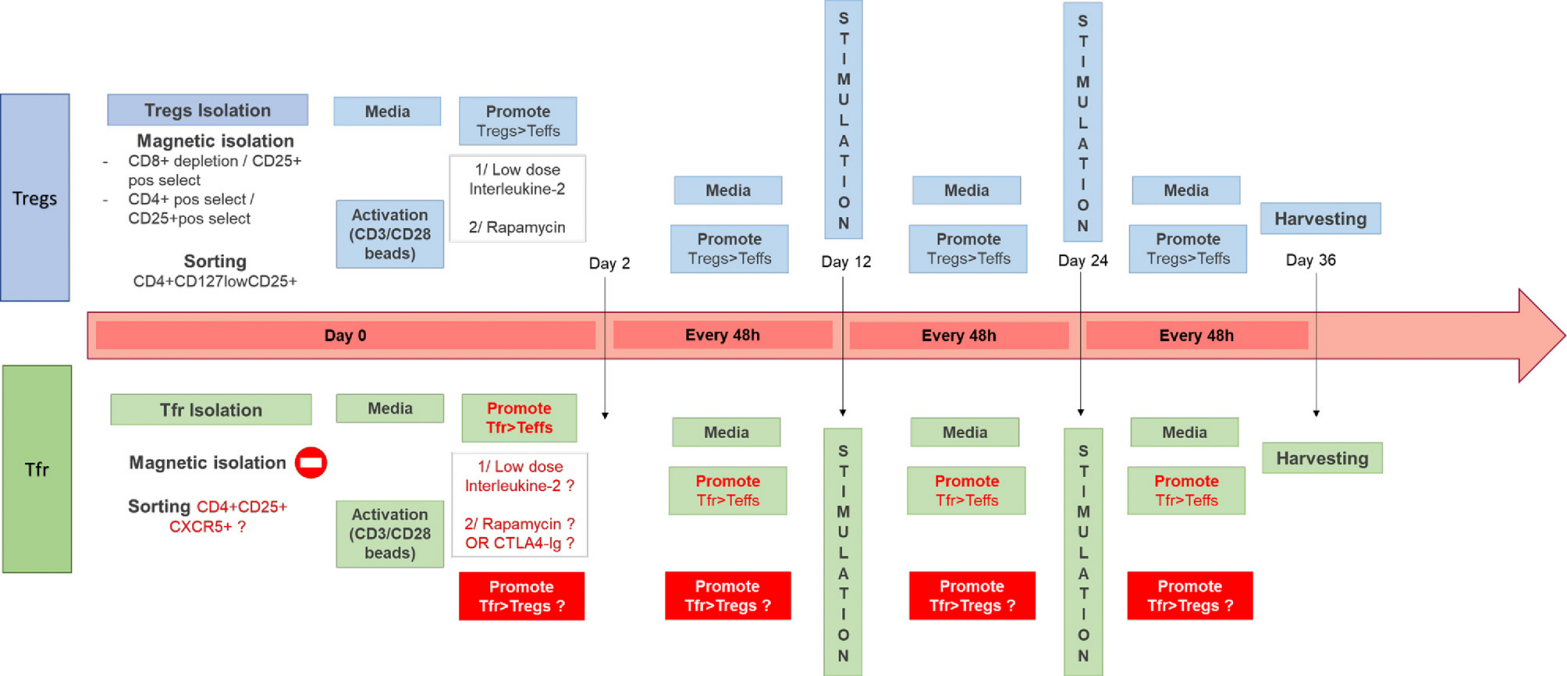
Different Tregs Good Manufacturing Product (GMP)-compatible expansion protocols and potential areas to improve for Tfr cell therapy. In the top diagram are described some of the protocols in use in our GMP facility (126, 127, 135) for the expansion of Tregs from the isolation step (using either magnetic beads or cell sorting) to the other steps such as stimulation every 12 days (with anti-CD3/CD28 beads) and feeding every 48 h. The use of IL-2 and rapamycin has been shown necessary to expand functional and stable Tregs in the absence of T effectors. In the bottom diagram is described a similar process for the generation of a Tfr product and in red are all the different steps which remains to be optimized.

The production and expansion of polyclonal autologous Tregs for cell therapy has been extensively described over the last few years, with a few protocol variations between centers (126, 127, 133–137). Others groups have been focusing on delivering autologous donor-antigen reactive Tregs to prevent transplant rejection (138, 139). In order to obtain a pure cell product which complies with Good Manufacturing Product (GMP) regulations before being reinfused to patients, experts have made a step forward standardization of required tests in a recent publication (140). The different options from isolation to culturing these cells are presented in **Figure 2**.

The communally accepted GMP-compliant process to expand polyclonal autologous Tregs developed in our laboratory which has been in use and demonstrated to be safe during the Phase I ONE Study (127) and ThRIL (126) includes harvesting Tregs from the (potential) recipient using CD8^+^ depletion and CD25^+^ positive selection (Miltenyi), followed by polyclonal expansion using CD3/CD28 beads (Dynabeads, Thermofisher) in the presence of Rapamycin (116, 135) and Interleukin(IL)-2 (141). This protocol allows the preferential expansion of Tregs compared to other T cell population. The aim of the expansion is to increase the numbers of cell present while maintaining phenotype, purity, and suppressive abilities. Other centers have used sorted cells as the initial Treg product (142), followed by expansion with or without Rapamycin present (138, 142, 143). As discussed previously the mTOR pathway seems to be essential for Tfr functions, therefore using Rapamycin to expand Tfr *ex vivo* might not be appropriate (86). Li et al. (144) demonstrated that patients on Sirolimus had lower numbers of CD4^+^CXCR5^+^ cells, but in this study Tfr and Tfh were not differentiated. Therefore, further exploratory work is needed before using Rapamycin in Tfr culture. Guinan et al. (139) have developed a technique using *ex vivo* costimulatory blockage with Belatacept to support the expansion of potent allo-specific Tregs. They perform a mixed lymphocyte reaction using T cells and allogenic PBMC stimulators in the presence of co-stimulatory blockade for 72 h. The cell product is then washed and the Treg fraction is isolated using CD8 and CD19 depletion, followed by CD25+ isolation prior to administration. These Tregs may represent induced Tregs, by co-stimulatory-deficient activation trough the TCR. This approach has been demonstrated to be safe, as part of the recently published ONE Study and is currently under investigation in a Phase I/II liver transplant study (NCT03577431). Using Belatacept to promote Tfr might be an attractive alternative to Rapamycin.

IL-2 is a crucial cytokine for T cell expansion. However, as Tregs constitutively express CD25, which is the α subunit of the IL-2 receptor, Treg proliferation requires less IL-2. Using low-dose IL-2 is therefore a way to foster Treg expansion in preference to Teffs, which require higher doses of IL-2 to expand. Low dose IL-2 has been used in all GMP-compatible Treg expansion protocols (126, 127, 134, 142) except in the antigen-specific Treg expansion protocol published by Guinan et al. mentioned above (where there is no expansion per se). The sensitivity of Tfr to IL-2 remains to be determined. As described earlier, low dose IL-2 in the context of GvHD seems to positively influence Tfr. On the other hand, some mouse and human (30, 56) bTfr may express lower levels of CD25 than other Tregs, and thus may not respond as well to lower doses of IL-2 compared to other Tregs. Moreover, high levels of IL-2 may be detrimental for Tfr expansion (30), as described by Botta et al. using a mouse model of influenza, although Tfr in this model seem to be differentially regulated by IL-2 during the early and later stages of infection. Therefore, the relevance and importance of low-dose IL-2 in protocols to isolate and expand autologous Tfr remains to be determined.

It remains to be explored if current protocols in place for Treg expansion could be extrapolated to Tfr expansion.

## Potential Applications in Transplantation

Despite having a non-clear potential diagnostic and prognostic applications in the transplant settings, cell therapy using Tfr could have a high potential both in prophylactic and therapeutic applications. Assuming the challenges associated with isolation and expansion of Tfr are solved, there are still multiple uncertainties regarding optimal indication (tolerance induction, treatment of CAD secondary to DSA), dose (in relation to number of Tfh), timing (in relation to the transplant and the need to re-dosing), and antigen specificity (to promote specific suppression of DSA production).

In summary, improving the understanding of Tfr-Tfh interactions might be the relevant to enhance long-term outcomes after transplantation. While Tfr have just started to be explored in the transplantation setting, their role remains to be further defined in human. Cell therapy using autologous Tfr has never been done, but should be technically feasible, with some refinement of existing protocols. Work to define the exact potential of such therapy is required.

## Author Contributions

CD participated in manuscript writing, editing, and coordination of its submission. SB contributed to manuscript writing. AD, GL, and CS contributed to manuscript writing and editing. AD and GL are co-last author of the manuscript. All authors contributed to the article and approved the submitted version.

## Funding

The authors have received funding from the Medical Research Council (MRC): award number MR/S000852/1 for CD and MR/T006560/1 for SB. This work was further supported by the Department of Health (DoH) *via* the National Institute for Health Research (NIHR) Comprehensive Biomedical Research Centre award to King’s Health Partners. Institutional Open Access funds to support article publication were also received. Views expressed are those of the authors and not necessarily those of the NHS, NIHR, or DoH.

## Conflict of Interest

GL is co-Founder of Quell Therapeutics.

The remaining authors declare that the research was conducted in the absence of any commercial or financial relationships that could be construed as a potential conflict of interest.
